# Tuberculosis transmission and community outreach screening in an informal urban settlement in a low-incidence European setting

**DOI:** 10.3389/fpubh.2026.1907926

**Published:** 2026-10-19

**Authors:** Clara Flamarich-Gol, Mar Bosch-Arís, Gloria Blázquez, Alicia Garcia-Circuns, Baye Elimane Thiaw, Hakima Ouaarab Essadek, Sílvia Roure, Zoran Stojanovic, Salomé Bellido-Calduch, Macarena Lovera, Alba García-Delgado, Antonia Segura, Anna Carreres-Molas, Ignasi Guasch-Arriaga, Adrià Antuori, Alba Llop, Pablo Pillado, Yolanda Romero, Olga Antolín, Jordi Gómez i Prat, Ignasi Parrón, Elsa Plasencia, Cristina Vilaplana

**Affiliations:** 1 Sant Roc Primary Care Center, Catalan Health Institute, Barcelona, Spain; 2 Research Support Unit Metropolitana Nord, Primary Care Research Institut Jordi Gol (IDIAPJGol), Mataró, Spain; 3 Departament of Medicine, Faculty of Medicine, Universitat Autònoma de Barcelona, Barcelona, Spain; 4 Epidemiological Surveillance Service of Barcelonès Nord and Maresme. Secretaria de Salut Pública. Departament de Salut. Generalitat de Catalunya., Barcelona, Spain; 5 Department of Infectious Diseases, Public Health and Community Team (eSPiC), Drassanes-Vall d’Hebron International Health Unit (USIDVH), Vall d’Hebron University Hospital, Barcelona, Spain; 6 International Health Unit of North Metropolitan Barcelona, Territorial Directorate for Infectious Diseases, Catalan Institute for Health, Badalona, Spain; 7 Fundació Lluita Contra les Infeccions, Badalona, Spain; 8 Germans Trias i Pujol Hospital and Research Institute (HUGTIP-IGTP), Badalona, Spain; 9 Microbiology Department, North Metropolitan Clinical Laboratory, Germans Trias i Pujol University Hospital, Badalona, Spain; 10 Centro de Investigación Biomédica en Red (CIBER) Enfermedades Respiratorias, Madrid, Spain

**Keywords:** immigration, primary health care, public health, social determinants of health, tuberculosis

## Abstract

**Background:**

Tuberculosis (TB) in low-incidence European settings disproportionately affects structurally vulnerable populations, including individuals living in informal housing. In late 2023, a TB cluster was identified among residents of an occupied building in Badalona, Spain. We report the implementation and outcomes of a community-based active TB screening intervention conducted in response.

**Methods:**

We conducted a descriptive field investigation between October 2024 and February 2025 in an occupied former school that served as a residence and social hub for approximately 150 individuals (residents and non-resident community members). Three on-site interventions combined health education, symptom-based screening, and clinical assessment. Individuals with TB-compatible symptoms were referred for chest radiography and microbiological testing (smear microscopy, culture, and Xpert MTB/RIF Ultra) according to national protocols. WHO-recommended screening cascade indicators were calculated. Additional TB cases epidemiologically linked to the settlement were identified through surveillance data.

**Findings:**

Eighty-one individuals were screened (54% of approximately 150 individuals connected to the settlement). Twelve presumptive TB cases were identified; all completed diagnostic evaluation. Three new TB cases were diagnosed through screening (number needed to screen = 27; number needed to test = 4). Two started treatment, and both completed it and were cured; the third never started treatment and was lost to follow-up. Overall, 11 TB cases were epidemiologically linked to the settlement (10 bacteriologically confirmed and one clinically diagnosed). The intervention also facilitated linkage to primary healthcare services. Operational challenges included high mobility, incomplete population enumeration, limited transport support, and institutional mistrust.

**Interpretation:**

Active TB transmission can persist in informal urban settlements in low-incidence settings. Community-based outreach screening is feasible but operationally fragile and dependent on timely identification, institutional coordination, and social support to ensure adequate coverage and continuity of care.

## Introduction

Tuberculosis (TB) is a disease of inequality. Despite unprecedented global progress, it continues to cluster in populations exposed to social and economic precarity. In 2024, an estimated 10.7 million people fell ill with TB and 1.23 million died from the disease; most of them in low -and middle- income countries, but also within vulnerable communities in high-income regions (1). According to WHO’s End TB Strategy, eliminating the disease by 2035 will require not only biomedical advances but also interventions addressing the social and structural determinants that drive exposure, infection, and delayed diagnosis (2, 3).

In Western Europe, TB incidence has steadily declined over recent decades, yet the disease remains entrenched among groups living at the margins of society: people experiencing homelessness, migrants in irregular situations, and those in overcrowded or informal housing (1). In 2020, more than one third of TB cases detected in the European Union or European Economic Area occurred in foreign-born individuals (4). Within these populations, the risk of transmission is amplified by poor living conditions, limited access to healthcare, and the fragmentation of social protection systems (5). The European Centre for Disease Prevention and Control (ECDC) and WHO have both called for targeted screening and integrated care models for “structurally vulnerable populations”, groups whose heightened risk of TB stems from the social conditions in which they live rather than from biological susceptibility (6, 7).

Spain is classified as a low-incidence country (<10 cases per 100,000 population) but continues to report localized clusters of transmission in urban areas marked by poverty and migration. In Catalonia, around two thirds of TB cases are detected among foreign-born individuals, and incidence rates in the most deprived districts of Barcelona are more than four times higher than in affluent areas (8). This pattern reflects broader inequalities in housing and employment, as well as persistent barriers to healthcare access despite universal coverage.

In late 2023, the Epidemiological Surveillance Service of Barcelonès Nord and Maresme (SVE BNM) detected a small cluster of TB cases among men of sub-Saharan origin living in Badalona, a densely populated city north of Barcelona. Epidemiological investigation linked several cases to a disused former secondary school building that had been occupied for 5 years as an informal housing settlement and housed approximately 150 people in precarious conditions. Shortly thereafter, another resident was hospitalized with disseminated TB and died during admission, suggesting possible ongoing transmission within the settlement.

The situation underscored a major operational challenge in TB control: how to reach and diagnose individuals living outside formal housing and administrative systems. Previous experiences in Catalonia and other European cities have shown that conventional contact tracing and clinic-based screening often fail in such settings, where trust in institutions is limited and population turnover is high (9). In response, WHO recommends flexible, community-based screening for active TB disease, implemented through multidisciplinary teams and supported by social interventions such as transport facilitation and community mediation (6).

Guided by these principles, the intervention was implemented jointly by the SVE BNM, Germans Trias i Pujol University Hospital (HUGTiP), the Sant Roc Primary Care Centre (CAP Sant Roc) and the eSPIC, the public health and community team of the Drassanes International Health Unit at Vall d’Hebron Hospital.

This article reports the design, implementation, and outcomes of the intervention. It situates the findings within the broader global health agenda on TB elimination and social inequality, highlighting how structural factors—housing exclusion, migration, and gender inequity -continue to shape the epidemiology of TB even in high-income, low-incidence settings.

## Methods

### Study design and objectives

We conducted a descriptive public health field investigation of a tuberculosis outbreak involving an occupied building in Badalona (Barcelona metropolitan area, Spain) between October 2024 and February 2025. The investigation consisted of three sequential on-site screening interventions coordinated by the Epidemiological SVE BNM in collaboration with primary care, hospital, and community teams.

The objectives were: (1) to identify undiagnosed active TB cases among residents and close contacts of the settlement, and (2) to evaluate the operational feasibility and outcomes of systematic field-based screening in a structurally vulnerable population, in accordance with WHO recommendations for active TB case finding in high-risk settings.

### Setting and population

The investigation took place in a former secondary school, which had been occupied for 5 years and housed approximately 150 people at the time of the first field visit. The building functioned not only as a residence but as a social hub for the surrounding community: beyond those living there, individuals attended regularly for social, religious and community gatherings. For screening purposes, the eligible target population was therefore defined as all individuals with a regular connection to the settlement -those residing in the building and non-resident individuals who used it regularly- rather than overnight residents alone. The estimate of approximately 150 individuals refers to this target population.

The building was divided into two zones A and B. Zone A consisted of a cluster of makeshift dwellings constructed with improvised materials, and zone B used the majority of building structure.

Living conditions were precarious, characterized by overcrowding, poor ventilation, makeshift partitions and limited access to water and electricity. Initial estimates of the resident population were uncertain, even the presence of women and children was unknown, requiring updates through successive visits. People who use drugs (cannabis and alcohol) were present in both areas.

### Community engagement and recruitment

Residents and other individuals present at the settlement were informed of the screening activity through posters, word of mouth, and on-site visits facilitated by community health workers (CHW) from eSPIC and other local non-governmental organizations (NGO). Trusted community representatives were identified to facilitate communication with residents and improve access to the community. All individuals present at each field visit were eligible, whether or not they resided in the building. Verbal consent was obtained before participation, and information was provided in accessible language and translation when needed.

### Screening procedures

Each field visit followed the same circuit (Figure 1). It began with a short health education talk on tuberculosis transmission, prevention and treatment, delivered on site. Personal identification and social data were then recorded, including country of origin, date of arrival in Europe, time living in the settlement, and language-related information. The screening questionnaire recorded whether a language barrier was present and, when applicable, its extent and the language spoken. When necessary, a community health worker or another person assisted with translation. The questionnaire then continued with a structured symptom questionnaire based on the WHO screening handbook covering unexplained cough and unexplained fever of more than 3 weeks, night sweats, dyspnea, chest pain, unintentional weight loss, unexplained fatigue, and date of symptom onset. Individuals who reported any symptom or were identified as close contacts of a TB case underwent an on-site clinical evaluation by a physician, during which symptoms were reassessed, general appearance and any visible abnormalities were evaluated, and information on major comorbidities, current medication, and relevant medical or surgical history was recorded. Close contacts were referred for chest radiography irrespective of symptoms. The full form is provided as Supplementary material.

**Figure 1:**
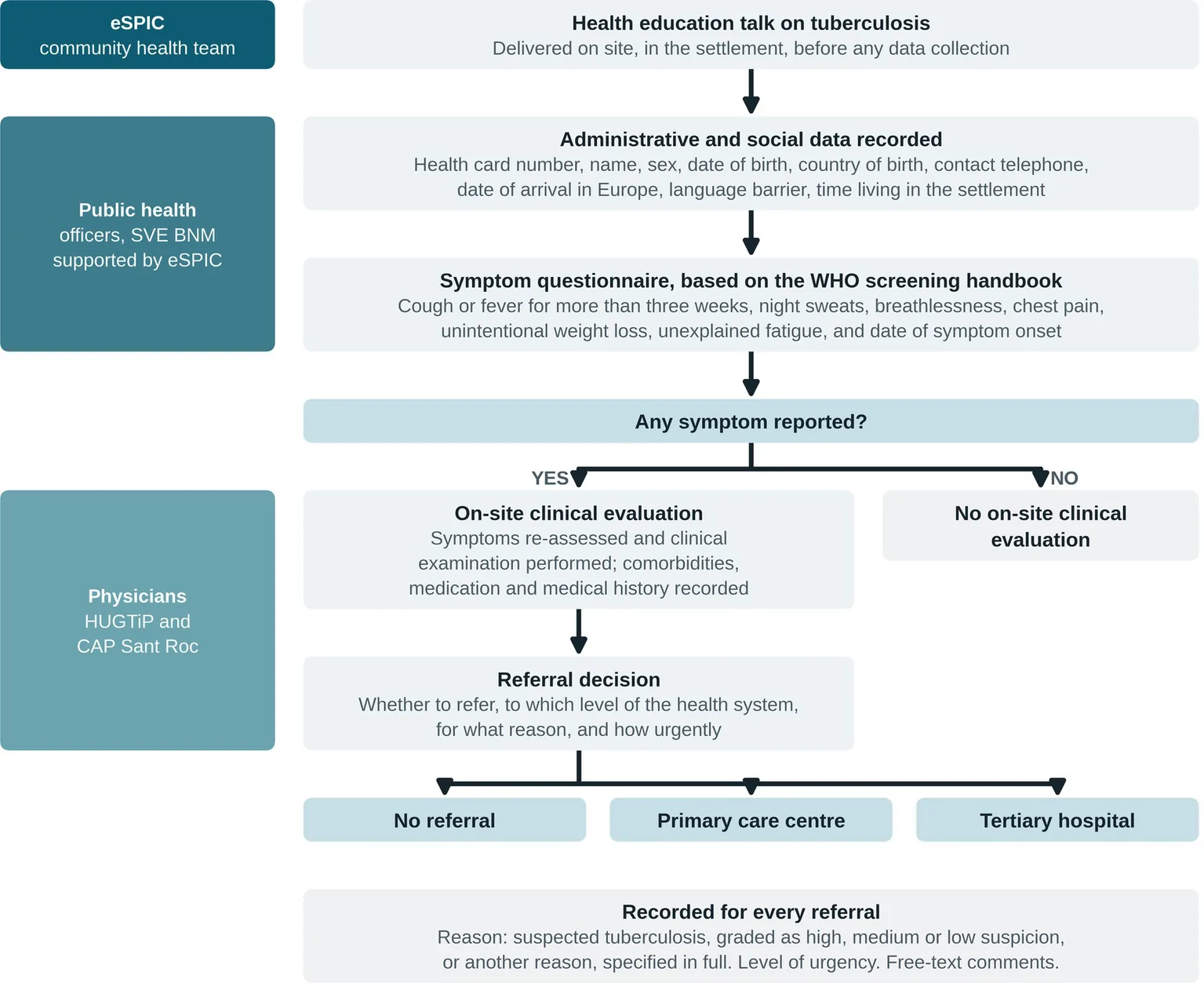
Field screening circuit. Sequence of steps applied at each on-site field visit, with the team responsible for each step shown on the left. Administrative and social data and the symptom questionnaire were completed for every participant. On-site clinical evaluation and the referral decision were carried out for those who reported any symptom or were identified as close contacts of a TB case, the latter being referred for chest radiography irrespective of symptoms. The symptom questions follow the WHO screening handbook. The full form is provided as Supplementary material.

On the basis of this evaluation, participants were classified as requiring no referral; referred to primary care for non-TB conditions or problems of health coverage; or referred to HUGTiP, either for suspected TB disease, graded as high, medium or low suspicion, or for non-respiratory conditions requiring hospital assessment. For every referral, the reason and the level of urgency were recorded. Referrals to HUGTiP included chest radiography, sputum smear and culture, and Xpert MTB/RIF Ultra testing, following national TB control protocols. Participants diagnosed with active TB were linked to the directly observed therapy (DOT) program.

### Facilitating access

Transportation cards were provided to referred individuals to ensure attendance at diagnostic facilities. Community health workers from eSPIC, collaborating NGO and the Germans Trias i Pujol Institute Foundation (IGTP) supported logistics and communication.

### Data collection and analysis

Data were collected using a structured field questionnaire (Supplementary material) and through a review of medical records from CAP Sant Roc and HUGTiP. *Screened* was defined as any individual who took part in a field intervention. *Clinically examined* was defined as any screened individual who completed both the symptom questionnaire and the on-site medical assessment. *Presumptive TB* was defined as any examined individual with TB-compatible symptoms or signs who was referred for diagnostic evaluation. Variables included: number eligible, screened, examined, referred, evaluated, and diagnosed.

WHO-recommended TB screening cascade indicators were calculated: *Acceptability* (screened/eligible), *Positivity* (presumptive/clinically examined), *Retention in testing* (evaluated/presumptive), *Number needed to screen (NNS)* = screened/diagnosed, *Number needed to test (NNT)* = evaluated/diagnosed.

Analyses were descriptive, expressed as frequencies and proportions. Data were aggregated across the three field interventions.

New registrations with the public health system among residents of the settlement were ascertained from the administrative records of CAP Sant Roc, identified through the settlement address recorded at registration. Individuals were counted only where no previous record existed in the system and where at least one attendance at the primary care center was documented. Each individual was counted once. Ascertainment covered the period from the start of the first field intervention to the time of writing and was therefore not restricted to the three screening visits.

### Ethical considerations

All data used in the analysis were collected during routine Public Health Surveillance activities as part of the legislated mandate of the Health Department of Catalonia, which is officially authorized to receive, process and temporarily store personal data in the case of a communicable infectious disease. For the present analysis, data were analyzed without direct personal identifiers. As all study activities formed part of statutory public health surveillance, Ethics Committee/Institutional Review Board review and individual informed consent were not required under the applicable legislation (10).

## Results

### Overview of interventions

Between October 2024 and February 2025, three field interventions were conducted at the occupied former institute. Approximately 150 individuals made up the target population connected to the settlement during the study (Table 1).

**Table 1:** Summary of field interventions.

Category	*N*	%
1st field intervention		
Estimated target population connected to settlement	150	–
Participants in intervention	55	36.6
Completed TB questionnaire and medical assessment	19	34.5
No referral required	3	15.8
Referred to primary care (non-TB symptoms)	9	47.4
Referred to HUGTiP for suspected TB	7	36.8
Attended HUGTiP	6	85.7
TB diagnoses among those evaluated	2	28.6
Seen at CAP (follow-up)	1	14.3
Non-referred individual examined voluntarily	1	–
2nd field intervention		
Participants in intervention	25	–
No referral required	2	8
Referred to CAP (non-TB conditions)	1	4
Referred to HUGTiP (non-respiratory symptoms)	4	16
Referred to HUGTiP (high suspicion of TB)	5	20
Referred to HUGTiP (asymptomatic close contacts)	13	52
Attended HUGTiP	15	—
Among those attending, previously referred	10	66.6
High suspicion of TB (examined)	2	13.3
Diagnosed TB cases	1	20
Non-referred but attended	5	33.3
Evaluated at CAP (high suspicion of TB)	3	–
3rd field intervention		
Women identified	12	–
Children identified	7	–
Participants in intervention	1	–
Women evaluated at CAP	1	8.3
Children evaluated	0	–

Percentages are calculated on the following denominators. First intervention: participants (*n* = 55) for “Completed TB questionnaire and medical assessment”; those completing assessment (*n* = 19) for the referral rows; and those referred to HUGTiP for suspected TB (*n* = 7) for “Attended HUGTiP,” “Seen at CAP” and “TB diagnoses among those evaluated.” Second intervention: participants (*n* = 25) for the referral rows; those attending HUGTiP (*n* = 15) for “Among those attending, previously referred,” “High suspicion of TB (examined)” and “Non-referred but attended”; and presumptive cases (*n* = 5) for “Diagnosed TB cases.” Third intervention: women identified (*n* = 12).

Across all activities, 81 people were screened, representing 54% acceptability among an estimated 150 eligible individuals (Table 2). Among the 45 people examined for TB symptoms, 12 presumptive TB cases were identified (positivity = 27%). All 12 presumptive TB cases completed diagnostic evaluation. Three were diagnosed with TB, giving a number needed to screen (NNS) of 27 and a number needed to test (NNT) of 4. Two of the three diagnosed patients, initiated treatment during the study period. Participants were individually identified at each field visit; cross-checking these identifiers confirmed that no individual took part in more than one intervention.

**Table 2:** WHO screening indicators.

WHO indicator	Value; % (when appropriate)
Eligible individuals for TB screening (*n*)	150
Individuals actually screened (*n*)	81
Individuals examined for TB symptoms (*n*)	45
Presumptive TB cases identified (*n*)	12
Individuals referred for suspected TB (*n*)	12
Individuals evaluated for TB disease (*n*)	12
Individuals diagnosed with TB (*n*)	3
Individuals started on TB treatment (*n*)	2
Acceptability (screened/eligible; %)	81/150; 54%
Positivity (presumptive/examined %)	12/45; 27%
Retention in testing (evaluated/presumptive; %)	12/12; 100%
Number needed to screen (NNS) (screened/diagnosed)	81/3; 27
Number needed to test (NNT) (evaluated/diagnosed)	12/3; 4
Individuals completing treatment (*n*)	2
Individuals never starting treatment, lost to follow-up (*n*)	1
Treatment success (completed/started; %)	2/2; 100%

WHO indicators as recommended by WHO consolidated guidelines on tuberculosis. Module 2: screening–systematic screening for tuberculosis disease. ^6^The denominator of 150 is an estimate of the target population connected to the settlement—those residing in the building and non-resident individuals who used it regularly for social, religious and community activities, including the women and children reached through community outreach. The third field intervention targeted women and children who spent time in the settlement without residing in it; of these, one woman was screened and is included in the numerator, and no children were screened. Of the 81 individuals screened, 80 resided in the building and one was the non-resident woman linked to the settlement; the numerator and denominator therefore refer to the same target population. N, number; TB, tuberculosis; %, percentage. Treatment outcomes were assessed up to April 2026.

### First field intervention

The first field activity, conducted in October 2024, reached 55 male participants (36.6% of the estimated target population). Nineteen completed full clinical assessments: three required no referral, nine were referred to primary care for unrelated conditions, and seven were referred to HUGTiP for suspected TB.

Six of the seven attended hospital appointments, where chest X-rays were performed. The person with suspected TB who did not attend the hospital was examined at the primary care center, obtaining a non-pathological chest X-ray. All seven presumptive cases were therefore evaluated. Two new cases of smear-positive pulmonary TB were confirmed (28.6% yield among those evaluated), both admitted for treatment and subsequently transferred to a specialized residential facility for DOT. One non-referred individual presented voluntarily and was also examined, with normal findings.

### Second field intervention

The second intervention in November 2024 aimed to expand coverage and include close contacts of confirmed cases. Twenty-five individuals participated (24 males and 1 woman). Five were referred to HUGTiP for TB-related symptoms and four for non-respiratory symptoms. Thirteen asymptomatic close contacts of the TB cases identified in the first intervention were also referred to HUGTiP for radiographic evaluation.

Fifteen attended HUGTiP, including 10 previously referred and five additional volunteers. Of the five presumptive cases, two were examined at HUGTiP and three were evaluated at the primary care center. One case of probable lymph node TB was diagnosed (20% yield among those evaluated), later lost to follow-up before treatment. Others had normal chest X-rays or non-TB findings.

### Third field intervention

The third visit, in February 2025, focused on women and children linked to the settlement. Despite identifying 12 women and 7 children, only one woman attended evaluation. She had been treated for TB years before and had a normal chest X-ray. No children were screened during the intervention.

### Aggregated outcomes

By the end of the investigation, 11 TB cases were epidemiologically linked to the settlement—10 bacteriologically confirmed and one clinically diagnosed- comprising: the initial index case, one fatal disseminated TB case, two new pulmonary TB cases detected during the first intervention and bacteriologically confirmed, one probable lymph node TB case from the second intervention, detected by chest X ray, and six additional cases identified through hospital and surveillance data. Two of the three individuals diagnosed through screening started treatment, both bacteriologically confirmed pulmonary cases; both completed treatment and were cured. The third, the probable lymph node TB case identified in the second intervention, never started treatment and has not been traced since. Treatment outcomes were assessed up to April 2026. Figure 2 summarizes the aggregated outcomes.

**Figure 2:**
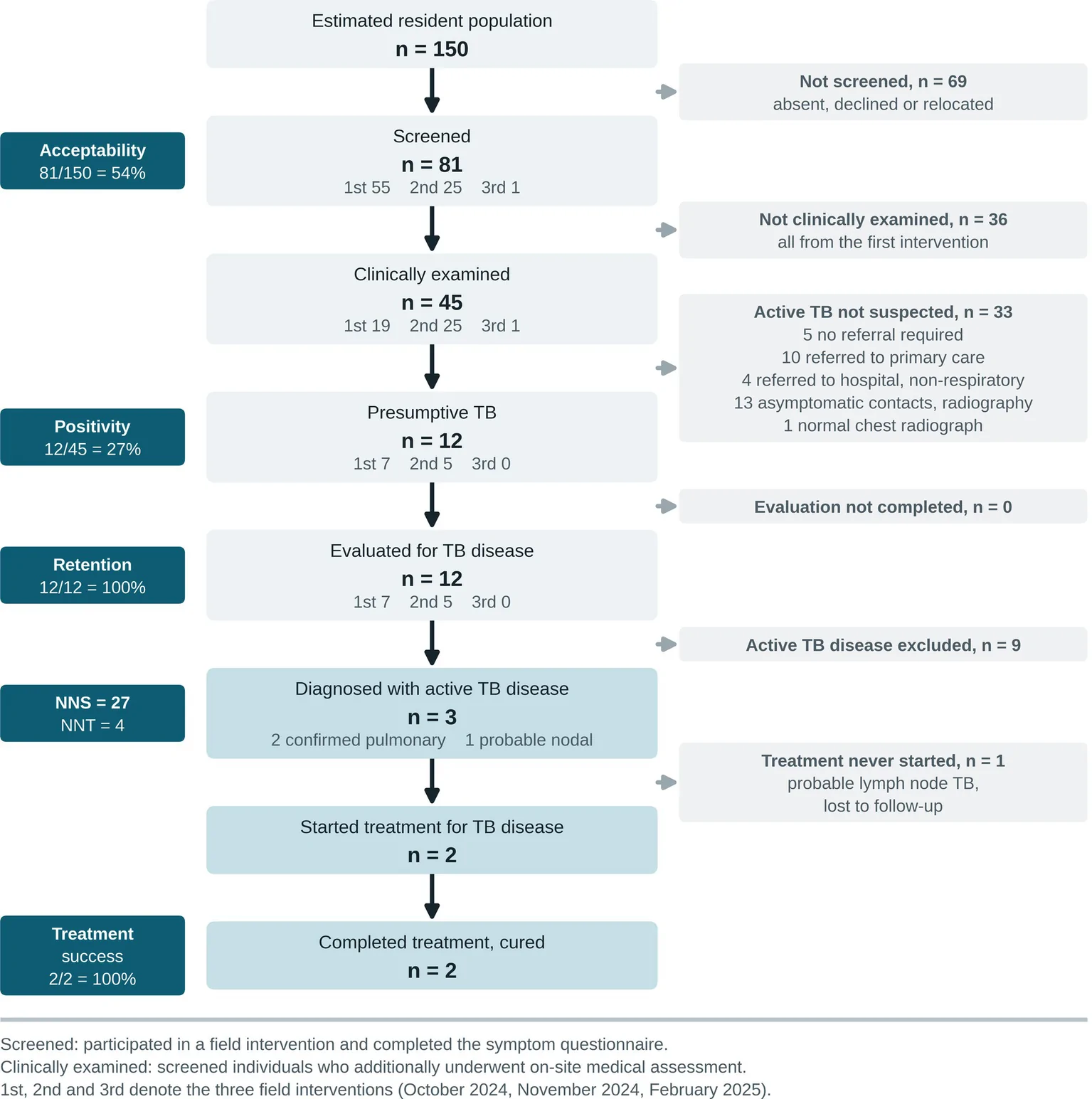
Screening cascade for active tuberculosis disease in an occupied former school in Badalona, Spain, October 2024–February 2025. The cascade refers throughout to screening for active TB disease; it does not describe testing for TB infection. Boxes on the central axis show the number of individuals at each step, with the contribution of each of the three field interventions; boxes on the right show the number not proceeding to the next step. Tags on the left show the WHO screening cascade indicators reported in Table 2. *Presumptive TB* denotes individuals with TB-compatible symptoms or signs referred for diagnostic evaluation; *active TB disease excluded* denotes individuals in whom diagnostic evaluation did not identify TB disease, irrespective of TB infection status. Among the 33 individuals in whom active TB was not suspected, 5 required no referral, 10 were referred to primary care for non-TB conditions, 4 were referred to hospital for non-respiratory symptoms, 13 were asymptomatic close contacts referred for radiographic evaluation and 1 was a previously treated woman with a normal chest radiograph. Treatment outcomes were assessed up to April 2026. TB, tuberculosis; NNS, number needed to screen; NNT, number needed to test.

### Operational insights

Participation was influenced by population mobility, distrust of institutions, and limited logistic support. Women and children were significantly underrepresented. Delays in site identification and incomplete baseline mapping reduced coverage. Data collection in this highly mobile and marginalized population was also limited, with incomplete personal information and fluctuating residency complicating accurate enumeration, follow-up, and evaluation of screening coverage. In addition, insufficient administrative support and the absence of systematic coordination with social services constrained the ability to address legal, housing, and documentation issues that directly affected healthcare access and continuity of care. Despite these challenges, the intervention demonstrated that coordinated, on-site screening -supported by hospital linkage and community mediation- can identify active TB cases in settings of extreme vulnerability.

During the ascertainment period, a further 66 residents of the settlement who were not screened for TB and had no previous record in the public health system registered with and attended CAP Sant Roc, in the context of the community engagement and referral activities associated with the intervention. Three children were also localized and confirmed linked to pediatric primary care for health needs unrelated to TB screening.

## Discussion

This field investigation describes one of the few documented tuberculosis screening interventions conducted in an occupied or informal housing facility in Western Europe. Among an estimated 150 eligible individuals, mainly men from sub-Saharan region, 81 were screened, 12 presumptive TB cases were identified, and three new cases were diagnosed. The campaign confirmed ongoing TB transmission in a highly marginalized community and demonstrated the feasibility of coordinated field-based screening despite major logistical and social barriers. Although diagnostic yield was moderate, the findings reveal how TB persists in small, socially excluded pockets within low-incidence countries. In such environments, the combination of precarious living conditions, mobility, and limited trust in institutions undermines both disease control and social protection mechanisms.

Active tuberculosis disease screening, as opposed to latent infection testing, remains a key strategy in low-incidence settings to identify and treat infectious cases among high-risk groups before transmission occurs. Systematic screening in these populations improves early diagnosis compared with reliance on passive case finding alone and may lead to better outcomes, including reduced mortality. It is therefore recommended even where direct evidence remains limited, as the potential benefits of early detection and prevention of severe outcomes outweigh potential harms (6, 11). However, programs must prioritize target groups carefully, since the evidence base on cost-effectiveness and long-term impact is still evolving (6, 12).

In Europe, the yield of active TB screening is generally highest among asylum seekers, refugees, and those migrating for family reunification, while it is lower among labor or student migrants (13). The consensus is that targeted migrant screening can detect active TB earlier in individuals who might otherwise be diagnosed later after arrival, thus improving prognosis and reducing onward transmission within host communities (6, 9). In low-incidence countries, screening yield in the general population tends to be low but rises substantially in high-risk groups, particularly among those originating from high-incidence countries. A recent systematic review suggests that targeted TB screening of migrants from countries with TB incidence rates of around 40 cases per 100,000 population or higher may be cost-effective (5).

Nevertheless, relying solely on country-of-origin TB incidence may be insufficient, as the risk among foreign-born individuals in Europe is highly heterogeneous and often exceeds national incidence figures. A high TB incidence rate in the country of origin does not always correlate with the risk faced by migrants after resettlement, which may be amplified by social vulnerability and living conditions. Therefore, screening strategies must be informed by local epidemiological data and consider differential risk factors to optimize effectiveness and cost-effectiveness (4, 13).

Screening programs must remain adaptable to demographic and geopolitical changes, including migration surges from conflict zones that may require rapid scaling of capacity. Belonging to certain migrant categories -such as asylum seekers or refugees- is consistently associated with higher screening yield, suggesting that conditions during migration, including overcrowded shelters and precarious travel routes, contribute to TB risk beyond the country-of-origin burden (13, 14).

Beyond the three interventions, the outbreak remains active as further cases of TB related to the settlement have been diagnosed. The settlement represents a structural hotspot, a setting where tuberculosis transmission is sustained not by individual risk factors but by systemic social exclusion. Recent evidence indicates that community-level socioeconomic indicators are often stronger predictors of TB case detection than historical notification rates, suggesting that under-detection has likely obscured the true disease burden in such environments. Integrating socioeconomic and spatial data into public health planning could therefore improve the identification of high-yield locations for targeted screening interventions (15).

These findings have implications for TB surveillance in low-incidence European settings. Informal or unstable housing environments may remain undetected by routine surveillance systems until multiple cases present, particularly when residents have limited interaction with formal healthcare services (4). In such contexts, conventional contact tracing and clinic-based case finding may be insufficient to identify ongoing transmission in a timely manner. Incorporating local socioeconomic and housing vulnerability indicators into surveillance assessments, and enabling rapid deployment of community-based outreach screening, may strengthen early detection and outbreak response among structurally vulnerable populations.

Three main operational barriers emerged during the outbreak response. First, site identification was delayed by 6 months after the initial reported cases, highlighting gaps in surveillance sensitivity and inter-institutional information flow. In addition, incomplete mapping and poor baseline data hindered accurate enumeration of the target population, making it difficult to estimate coverage and resource needs and resulting in under-screening of certain building zones. For this reason, four extra months were required to perform the first intervention. Finally, logistical and social constraints -including lack of dedicated transport funding, heavy reliance on NGO support, and limited on-site accompaniment-significantly reduced referral adherence. Indeed, structural barriers such as transportation gaps, housing instability, and institutional mistrust can severely compromise screening outcomes. To address these, program designs must integrate social protection and people-centered support -such as transport subsidies and community-led accompaniment- as advocated by the WHO to effectively mitigate structural inequities (6, 16).

Gender-related disparities were evident in participation. Although women and children did not reside within the occupied building itself, the third screening round specifically targeted them through community outreach. Yet almost none attended. Healthcare professionals working in the field (including public health agents and primary healthcare workers) informally reported that some women were concerned that contact with public health services could attract the attention of social services. This highlights how structural vulnerability and institutional mistrust intersect with gender (17–20), creating additional structural barriers to TB screening, diagnosis, and continuity of care. Future interventions must therefore integrate gender-sensitive approaches that prioritize safety, confidentiality, and trust-building to ensure equitable access for women and families, as noted in recent community-based TB work in Barcelona which emphasize that addressing social determinants and legal fears is essential for equitable care (14).

A legal and ethical dimension must also be considered. It is the collective responsibility of public health authorities and implementing teams to ensure that tuberculosis screening -particularly among vulnerable populations - is exclusively used to improve population health and prevent transmission. Screening results should be used strictly for healthcare and epidemiological purposes, and all activities must be conducted with cultural sensitivity, confidentiality, and respect for participants’ dignity to prevent stigma or racism (6, 14).

This investigation did not include on-site chest X-ray screening and this is a limitation. Instead, participants with symptoms were referred to healthcare facilities for radiological evaluation, potentially introducing a risk of diagnostic attrition. However, all 12 participants with presumptive TB completed the diagnostic evaluation, and no diagnostic attrition was observed in our study. Although mobile chest X-ray units equipped with computer-aided detection (CAD) software are endorsed by WHO and enable rapid, high-throughput TB screening (21), their implementation remains constrained in many routine public health settings, where access is often limited outside externally funded projects. In most public health systems, such tools are not routinely accessible for field interventions of this kind, limiting cost-effectiveness and scalability.

Beyond case detection, the intervention implemented by the CHW yielded wider public health benefits. Community-based education activities improved residents’ understanding of tuberculosis transmission, symptoms, and treatment, helping to reduce misinformation and stigma (22, 23). Moreover, the intervention helped to successfully link some individuals to primary healthcare services for the first time (22, 23). The settlement was also identified as a dynamic transmission hotspot, prompting additional field interventions, community activities, and active TB screening in a neighboring occupied facility.

A major strength of this study was the effective intersectoral collaboration established between epidemiological, clinical, primary care, and community teams. Evidence from implementation science shows that TB screening among socially vulnerable populations depends on partnerships that extend beyond conventional health services (14, 24). The intervention also succeeded in building trust with residents of the occupied building, linking them to the public health system not only for TB diagnosis and care but also for broader health needs. This sustained engagement with a highly marginalized community represents the most important achievement of the intervention (25).

Several operational lessons emerged across levels of care. Conducting screening outside formal healthcare facilities proved substantially more complex, requiring detailed knowledge of the physical space, identification of trusted community leaders, and the creation of safe areas for interviews and clinical assessment.

Screening in the settlement was symptom-based and directed at active TB disease. Systematic testing for TB infection, with the offer of preventive treatment to close contacts as recommended by WHO, was considered but deliberately not included in the intervention, as it is often difficult to implement in settings of this kind. Populations in informal settlements are highly mobile, moving repeatedly for work and other circumstances; administrative status is often precarious; and individuals may live with considerable fear of contact with institutions. Together, these factors considerably complicate the implementation and continuity of a preventive care pathway.

Prioritizing symptomatic individuals within a sequential screening algorithm was essential given limited resources. Transportation support and direct accompaniment to healthcare facilities were critical to maintaining engagement, while providing clear information about healthcare rights- including access regardless of documentation status- helped reduce fear and improve participation. Completion of diagnostic evaluation was 100%, which we attribute primarily to the adaptation of referral pathways when necessary; for example, by offering assessment in primary care after a missed hospital appointment or evaluating individuals who presented without a formal referral. At the hospital level, the intervention strengthened coordination across emergency, respiratory, infectious disease, radiology, microbiology, and administrative services, leading to improved referral circuits and refinement of internal diagnostic algorithms for future outreach activities. Primary care involvement proved indispensable for recovering missed cases, addressing comorbidities, supporting administrative processes, and coordinating with social services, reinforcing the importance of community-oriented primary healthcare in TB control among vulnerable populations. Although services cannot adapt to every individual circumstance without increasing implementation complexity, a pragmatic balance between flexibility and operational simplicity may substantially reduce loss to follow-up among populations experiencing unstable housing.

Beyond immediate outbreak control, our findings highlight the need for institutionalized funding mechanisms for outreach-based TB screening, stronger data sharing between surveillance and social services, and the integration of screening activities into broader health and social protection frameworks. The field intervention also revealed how social and political dynamics can undermine public health efforts. During the course of the screening, an eviction notice was issued for the occupied building, prompting many residents to leave or relocate. Some individuals were consequently lost to follow-up and remained unscreened, underscoring that eviction processes should never be conducted during an ongoing health intervention, as they compromise both public health objectives and human rights.

Furthermore, the detection of TB cases and the intervention itself became a political issue, publicly discussed. Media coverage of the event, while raising awareness, may also have reinforced stigma and distrust toward vulnerable groups. These experiences demonstrate the importance of maintaining confidentiality and depoliticizing health actions to protect affected communities.

Finally, further research is needed to assess the cost-effectiveness and long-term impact of targeted active TB screening in urban informal settlements, and to identify optimal combinations of clinical, epidemiological, and socioeconomic indicators for case finding and resource allocation.

In conclusion, this investigation demonstrates that tuberculosis transmission can persist within informal urban settlements in low-incidence settings. Screening in such environments is feasible but operationally fragile, as it depends on trust, stability, and sustained institutional coordination. Housing instability, including eviction processes, and the public politicization of outbreaks may disrupt participation and continuity of care, potentially compromising outbreak control efforts. Effective active case finding in structurally vulnerable populations therefore requires implementation safeguards that account for these contextual risks.

## Data Availability

The original contributions presented in the study are included in the article/Supplementary material, further inquiries can be directed to the corresponding author.
