# Supplementary material for: Tuberculosis transmission and community outreach screening in an informal urban settlement in a low-incidence European setting
Source: Front Public Health. 2026 Oct 19;14:1907926. doi: 10.3389/fpubh.2026.1907926 (PMC13725769; doi:10.3389/fpubh.2026.1907926)
Supplement: Supplementary file 1 [file Data_Sheet_1.PDF]

Supplementary Material

Field screening questionnaire for tuberculosis

Community outreach screening in an informal urban settlement

This is the form used during the on-site field visits described in the manuscript. Sections 1 and 2 were completed for every person who agreed to take part; Sections 3 and 4 were completed only when one or more symptoms were reported in Section 2. The symptom questions follow the WHO screening handbook. The form is reproduced here in full so that it can be adapted and reused by teams working in comparable settings; it was administered in the field in Catalan, Spanish, English and French, with interpretation where required.

1 Administrative and social data

Health card number	
Name and surname(s)	
Sex	☐ Male ☐ Female ☐ Non-binary
Date of birth	
Country of birth	
Contact telephone	
Date of arrival in Europe	
Language barrier	☐ Yes ☐ No
Time living at this site	

Contact telephone: if the person has no telephone of their own, record the telephone of a contact. Where there is a language barrier, record the telephone of someone the person considers relevant to them.

2 Tuberculosis symptom questionnaire

Based on the WHO screening handbook. Ask every question, even when the person has already answered "no" to the first.

Has the person had any symptoms?	☐ Yes	☐ No
Unexplained cough for more than three weeks	☐ Yes	☐ No
Unexplained fever for more than three weeks	☐ Yes	☐ No
Night sweats (sweating that soaks the bedclothes)	☐ Yes	☐ No
Difficulty breathing	☐ Yes	☐ No
Chest pain	☐ Yes	☐ No
Unintentional weight loss	☐ Yes	☐ No
Unexplained fatigue (very tired for no particular reason)	☐ Yes	☐ No
When did these symptoms begin?		

3 On-site clinical evaluation

Completed on site only when one or more symptoms are reported in Section 2. Symptoms are re-assessed before examination.

Symptoms on re-assessment

Findings on clinical examination

Major comorbidities

Current medication

Relevant medical or surgical history

4 Referral decision

Is referral required? ☐ Yes ☐ No

If yes, to which level of the health system? ☐ Primary healthcare centre ☐ Tertiary hospital

Reason for referral

☐ Suspected tuberculosis — degree of suspicion ☐ High ☐ Medium ☐ Low

Relevant information supporting the suspicion

☐ Other reason — specify in full

Urgency of the referral

Comments
